# What factors influence cellular pathologists’ confidence in case reporting?

**DOI:** 10.1007/s00428-024-03899-1

**Published:** 2024-08-17

**Authors:** Harriet Evans, Peter K. Kimani, Louise Hiller, Yee Wah Tsang, Shatrughan Sah, Kishore Gopalakrishnan, Clinton Boyd, Maurice B. Loughrey, Paul J. Kelly, David P. Boyle, David Clark, Ian O. Ellis, Mohammad Ilyas, Emad Rakha, Adam Bickers, Ian S. D. Roberts, Maria F. Soares, Desley A. H. Neil, Janet A. Dunn, Ayesha Azam, David Snead

**Affiliations:** 1 Histopathology Department, University Hospitals Coventry and Warwickshire NHS Trust, Clifford Bridge Road, Coventry, CV2 2DX UK; 2 Warwick Medical School, University of Warwick, Coventry, UK; 3 Clinical Trials Unit, University of Warwick, Coventry, UK; 4 Belfast Health and Social Care Trust, Belfast, UK; 5 Queen’s University, Belfast, UK; 6 Nottingham University Hospital NHS Trust, Nottingham, UK; 7 University of Nottingham, Nottingham, UK; 8 Hamad Medical Corporation, Doha, Qatar; 9 Northern Lincolnshire and Goole NHS Foundation Trust, Scunthorpe, UK; 10 Oxford University Hospitals NHS Foundation Trust, Oxford, UK; 11 Birmingham NHS Foundation Trust, Birmingham, UK; 12 Institute of Immunology and Immunotherapy, University of Birmingham, Birmingham, UK; 13 Computer Science Department, University of Warwick, Coventry, UK

**Keywords:** Diagnostic confidence, Digital pathology, Gastrointestinal pathology, Skin pathology, Breast pathology, Renal pathology

## Abstract

Histopathology is a challenging interpretive discipline, and the level of confidence a pathologist has in their diagnosis is known to vary, which is conveyed descriptively in pathology reports. There has been little study to accurately quantify pathologists’ diagnostic confidence or the factors that influence it. In this study involving sixteen pathologists from six NHS trusts, we assessed diagnostic confidence across multiple variables and four specialties. Each case was reported by four pathologists, with each pathologist reporting each case twice (on light microscopy (LM) and digital pathology (DP)). For each diagnosis, pathologists recorded their confidence on a 7-point Likert scale. This provided 16,187 diagnoses and associated confidence scores for analysis. All variables investigated were found to be significantly predictive of diagnostic confidence, except level of pathologist experience. Confidence was lower for difficult to report cases, cases where there was inter- and intra-pathologist variation in the diagnosis, and cases where the pathologist made an incorrect diagnosis. Confidence was higher, although nominally, for LM diagnoses than DP (rate ratio 1.09 (95% CI 1.01–1.18), *p* = 0.035), although results indicate pathologists are confident to report on DP. Lowest confidence scores were seen in areas of known diagnostic complexity and cases with quality issues. High confidence in incorrect diagnoses were almost invariably attributed to interpretive diagnostic differences which occurred across both rare and common lesions. The results highlight the value of external quality control schemes and the benefits of selective peer review when reporting.

**Supplementary Information:**

The online version contains supplementary material available at 10.1007/s00428-024-03899-1.

## Introduction

A histopathology report represents an informed opinion made by a pathologist following assessment of a case [1]. Due to the subjective and interpretative nature of histopathological findings, which comprise a continuous scale on a biological-morphological spectrum, a pathologist’s confidence in the reports they issue will inevitably vary. Even the most experienced pathologists sometimes face uncertainty about the most appropriate classification of some pathological features they encounter. This level of uncertainty, or confidence, is often conveyed descriptively in the pathology report to allow the clinical team to make appropriate decisions regarding further patient management [2, 3].

Numerous factors govern why a pathologist may not be able to reach a confident definitive diagnosis, including a lack of clinical information, unusual presentation, rare or complex morphology, mismatch between morphology and immunohistochemical (IHC) profile, a lack of clear diagnostic criteria, inadequate material, a lack of experience or a desire to avoid legal liability from medical error [2–4].

Although it has been demonstrated that 35% of surgical pathology reports contain some expression of uncertainty [4], little is known about the degree of pathologists’ confidence in their diagnostic reporting or how confidence varies with different factors such as case type, speciality, level of experience or diagnostic modality. Understanding pathologists’ diagnostic confidence gives insight into the profession, could help to identify areas requiring training or colleague support, and serves as a baseline for comparison of novel modalities and diagnostic tools. It is particularly important to consider diagnostic confidence between light microscopy (LM) and digital pathology (DP) at this time of change in histopathology practice, to ensure that pathologists are confident with this new way of working. Although numerous validation studies demonstrate the high diagnostic concordance of DP to LM [5], successful implementation of DP also depends on individual pathologists’ uptake, support and, above all, confidence in using the technology [6].

This study provides a unique focus on pathologists’ confidence in case reporting and how this varies across numerous variables. This gives a novel insight into the profession and reporting practices, which is relevant to pathologists but also more widely to all who base patient care decisions on histopathology reports.

## Methods

### Study design

The data were collected as part of the multicentre National Institute of Health and Care Research (NIHR) funded Digital Pathology study [7]. The study included 608 breast, 607 gastrointestinal (GI), 609 skin and 200 renal cases enrolled between July 2019 to July 2021, from 5 NHS sites (Belfast, Coventry, Lincoln, Nottingham and Oxford). All available stains for a case (including haematoxylin and eosin (H&E), special stains, IHC and immunofluorescence) were included except for GI where only H&E were included. For biopsies, all slides were included, but for some large (> 10 block) resections, representative slides were selected that were sufficient to report the case [7]. Scanned whole slide images (WSI) were equivalent to × 40 magnification for review.

Sixteen pathologists (four from each speciality) from six NHS pathology departments reported the cases. Each pathologist reported all cases in their specialty twice, once using LM and once using DP, with a 6-week washout period between, and the order of LM and DP randomly assigned. At each read, pathologists recorded their confidence on a 7-point Likert scale, with 1 being the lowest and 7 being the highest confidence scores. This resulted in 16,192 diagnoses, with 16,187 corresponding confidence scores (confidence scores were missing for five diagnoses). Any differences in diagnosis were sent to an independent arbitration team, including clinicians, to decide if differences were likely to result in different patient management, i.e. clinically important difference (CID) or not, i.e. clinically unimportant difference (CUD).

The primary aim was to determine predictors of diagnostic confidence. Seven variables were assessed:

- Pathologist overall reporting experience (number of years a pathologist had been practising as a consultant: 3–10 years, 10.5–20 years and 21–35 years)
- Pathologist DP reporting experience (number of years they had reported on DP in routine practice: none, ≤ 1.5 years or 1.5–5 years)
- Modality on which diagnosis was made (LM/DP)
- Case specialty (breast/GI/skin/renal)
- Case difficulty level (routine/moderately difficult to report/difficult to report), based on the type of pathology present and specimen type (7)
- Whether a report’s diagnosis agreed with the ground truth (GT) diagnosis (defined in consensus meetings by reporting pathologists) (complete agreement (CA)/CID/CUD)
- Whether the LM report diagnosis agreed with the DP report diagnosis (CA/CID/CUD)

### Statistical analysis

Overall and DP reporting experiences were summarised in a scatter plot. For the other potential predictors of diagnostic confidence, number and percentage in each category were reported.

Stacked bar charts illustrate the confidence scores across the seven possible predictor variables. To fit statistical models, diagnostic confidence scores were analysed as count data because, with seven possible scores only, they could not be assumed to be normally distributed. This was done after inverting the scores from 1–7 to 6–0 so that zero (0) corresponded to the highest confidence and six (6) corresponded to the lowest confidence. This conversion allowed fitting zero-inflated Poisson models if necessary because a large majority of the diagnoses had the highest confidence rating. The variance of the raw scores was smaller than the mean of the raw scores, implying that the data were less dispersed than expected for standard Poisson count data. Therefore, accounting for each case being reported by four pathologists and a pathologist reading each case twice, a random effects (RE) generalised Poisson model with crossed RE terms for case and pathologist was used to determine the predictors of diagnostic confidence score. The model was fitted in R statistical program [8] using the “glmmTMB” package [9] and specifying the Conway–Maxwell–Poisson distribution. The parameterisation used allowed the comparison of mean rates. The model fitted with all confidence data scores did not include DP reporting experience because it was considered not logical to expect DP reporting experience to predict diagnostic confidence for LM reports.

In subgroup analysis, LM reports and DP reports were analysed separately. Two models were fitted for DP reports, excluding and including DP reporting experience. The model excluding DP reporting experience was to compare LM and DP models with common variables.

Qualitative analysis was performed on cases with an original confidence score of 1–3, to explore factors contributing to low confidence. Investigation was also undertaken into cases with both a high confidence (an original score of 7) and CID when compared to the GT diagnosis. These are potentially concerning scenarios where there is a diagnostic discrepancy, but the pathologist is highly confident. These cases were reviewed by a pathologist and the nature of the CID categorised. Cases where there was category uncertainty were discussed between three pathologists to decide on the most appropriate classification.

## Results

### Predictor variables

**Fig. 1:**
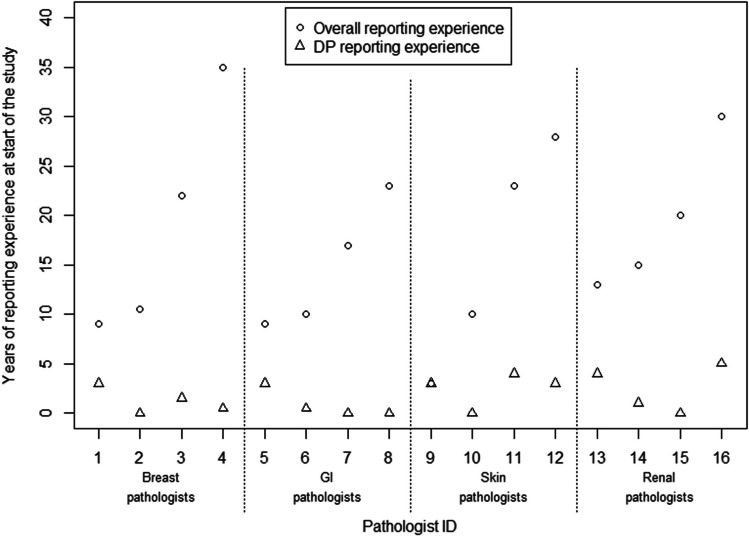
Pathologists’ reporting experiences

The overall reporting experience of the 16 pathologists was between 3 and 35 years. In terms of routine DP reporting experience, five pathologists had no experience, with the rest having up to 5 years of experience (Fig. 1).

71.5% of cases were considered routine, 8.1% moderately difficult and 20.4% difficult to report cases. Of the 16,187 diagnoses with corresponding confidence scores, 89% of diagnoses showed complete agreement with GT, whilst in 4.8% of the reports, there was CID between a proffered diagnosis and GT diagnosis. There was complete agreement between LM and DP diagnoses for 91% of the diagnoses and CID in 4% of the diagnoses.

### Diagnostic confidence

**Fig. 2:**
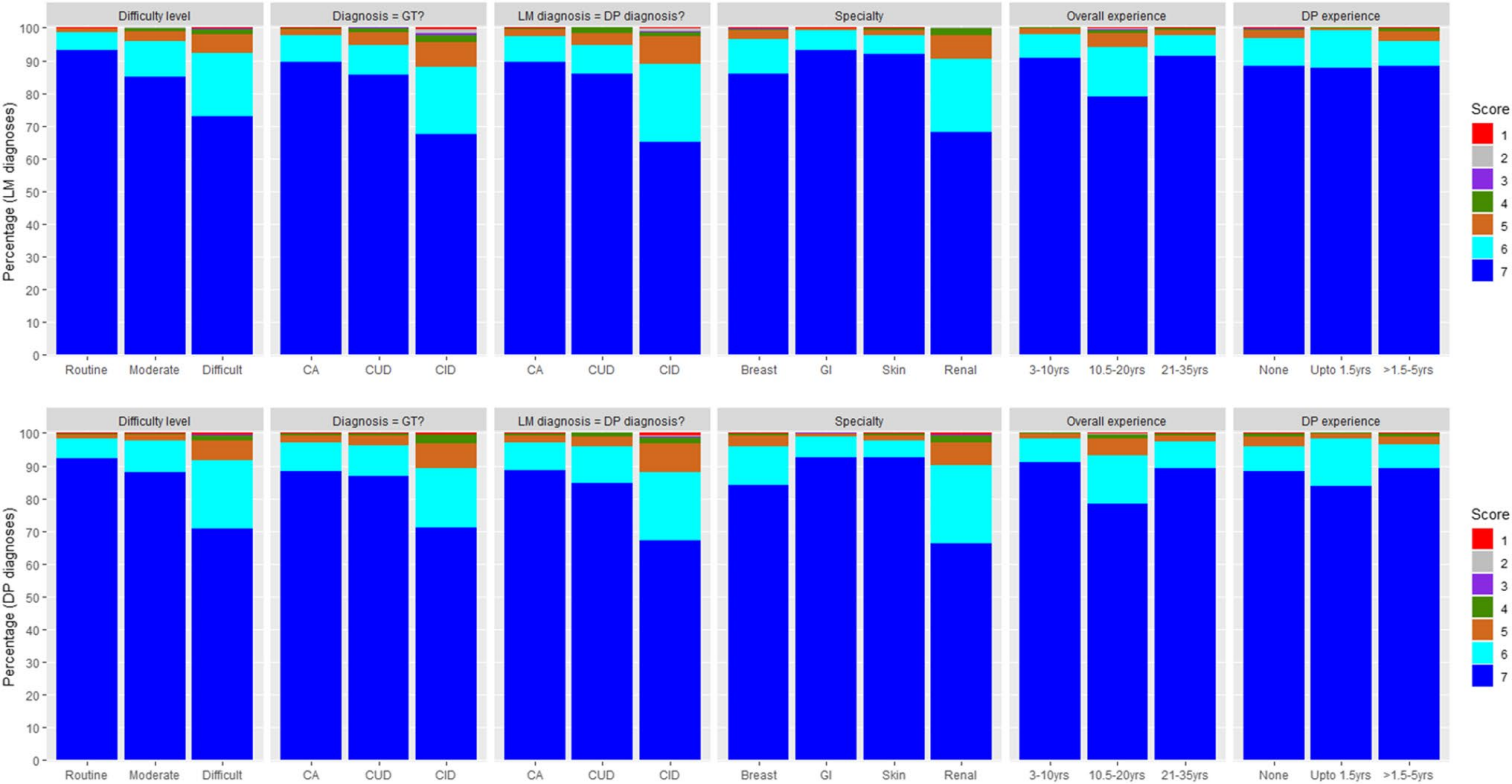
Percentage of different diagnostic confidence scores for LM (top row) and DP (bottom row) diagnoses in different categories of the potential predictor variables. Each score has an assigned colour, with dark blue corresponding to the highest diagnostic confidence score of 7. CA, complete agreement; CUD, clinically unimportant difference; CID, clinically important difference; yrs, years

Figure 2 shows the confidence scores for LM (top row) and DP (bottom row), for a range of variables (1 = lowest confidence score, 7 = highest confidence score). Overall, we see high confidence, with most diagnoses given one of the two highest scores. Confidence was slightly higher for LM diagnoses than for DP. As expected, for LM diagnoses, there was no relationship between diagnostic confidence and DP reporting experience (top right graph).

Similar trends were observed within the DP and LM modalities in terms of the relationship between confidence and the predictors. Confidence was lower for difficult to report cases when compared to routine and moderately difficult cases. Confidence was also noticeably lower when there was a CID between a pathologist’s diagnosis and GT or when there was a CID between LM and DP diagnosis (i.e. in cases where inter- and intra-observer diagnostic discrepancies existed). In comparison to pathologists with least experience (3–10 years), pathologists with moderate experience (10.5–20 years) are less confident, whilst the difference with pathologists with the most experience (21–35 years) is very small.

Over all data, all variables investigated were found to be significantly predictive of diagnostic confidence (Table 1). Diagnostic confidence was lower for DP reporting than LM reporting (rate ratio 1.09 (95% CI 1.01–1.18), *p* = 0.035). Diagnostic confidence was highest for routine cases and lowest for difficult to report cases (*p* < 0.001). Compared to when there is CA between a report’s diagnosis and GT, diagnostic confidence was lower when there is a CID (*p* = 0.002) or when there is a CUD (*p* < 0.001) with confidence lowest for the latter. Compared to where pathologists LM and DP diagnoses CA, diagnostic confidence was significantly lower when there was CUD between LM and DP diagnoses but not significantly lower for CID, indicating pathologists can still be confident when they give a different diagnosis for a case they have reported previously (i.e. in instances of intra-observer variability on multiple assessments of the case). As reported previously, there is high LM-DP intra-observer agreement for the cases in this study [7].

**Table 1:** Assessing predictors for diagnostic confidence using random effects generalised Poisson models

Parameter	Rate ratio† (95% confidence interval), *p*-value			
	All data	LM data only	DP data only	DP data only
			Excluding DP reporting experience in model	Including DP reporting experience in model
Intercept*	0.045 (0.028–0.073), < 0.001	0.052 (0.034–0.081), < 0.001	0.062 (0.039–0.100), < 0.001	0.055 (0.029–0.105), < 0.001
Modality (reference, LM) DP	1.09 (1.01–1.18), 0.035	NA	NA	NA
Difficulty level (reference, routine) Moderate Difficult	< 0.001^‡^ 2.01 (1.53–2.64), < 0.001 4.15 (3.31–5.21), < 0.001	< 0.001^‡^ 2.07 (1.56–2.76), < 0.001 3.66 (2.89–4.63), < 0.001	< 0.001^‡^ 1.41 (1.05–1.90), 0.023 3.51 (2.80–4.41), < 0.001	< 0.001^‡^ 1.41 (1.05–1.90), 0.023 3.52 (2.80–4.41), < 0.001
Diagnosis = GT (reference, CA) Clinically important difference Clinically unimportant difference	< 0.001^‡^ 1.38 (1.13–1.68), 0.002 2.23 (1.90–2.62), < 0.001	< 0.001^‡^ 1.65 (1.24–2.19), 0.001 2.39 (1.88–3.02), < 0.001	< 0.001^‡^ 1.25 (0.94–1.66), 0.125 2.63 (2.08–3.33), < 0.001	< 0.001^‡^ 1.25 (0.94–1.66), 0.123 2.63 (2.08–3.33), < 0.001
LM diagnosis = DP diagnosis (reference, CA) Clinically important difference Clinically unimportant difference	< 0.001^‡^ 1.18 (0.95–1.47), 0.138 1.89 (1.58–2.27), < 0.001	< 0.001^‡^ 1.19 (0.87–1.61), 0.274 2.10 (1.64–2.68), < 0.001	< 0.001^‡^ 1.20 (0.89–1.62), 0.239 1.79 (1.40–2.30), < 0.001	< 0.001^‡^ 1.20 (0.89–1.62), 0.238 1.79 (1.40–2.30), < 0.001
Specialty (reference, breast) GI Skin Renal	0.001^‡^ 0.35 (0.21–0.59), < 0.001 0.52 (0.31–0.89), 0.016 0.76 (0.42–1.39), 0.380	0.001^‡^ 0.38 (0.24–0.61), < 0.001 0.71 (0.44–1.14), 0.152 0.83 (0.48–1.43), 0.504	0.002^‡^ 0.38 (0.23–0.64), < 0.001 0.55 (0.33–0.92), 0.023 0.81 (0.45–1.46), 0.480	0.008^‡^ 0.40 (0.24–0.68), 0.001 0.59 (0.34–1.04), 0.068 0.80 (0.43–1.49), 0.485
Overall reporting experience (reference, 3–10 years) 10.5–20 years 22–35 years	0.019^‡^ 2.05 (1.19–3.53), 0.009 1.12 (0.73–1.73), 0.604	0.002^‡^ 1.99 (1.21–3.25), 0.006 0.94 (0.63–1.40), 0.759	0.060^‡^ 1.90 (1.11–3.28), 0.020 1.21 (0.78–1.88), 0.385	0.083^‡^ 2.04 (1.08–3.85), 0.028 1.21 (0.78–1.87), 0.394
DP reporting experience (reference, none) > 0–1.5 years 3–5 years	NA	NA	Not included to have a similar model to compare with the model for LM data	0.784^‡^ 1.20 (0.71–2.04), 0.497 1.07 (0.64–1.77), 0.801

^†^Rate ratio unless otherwise stated—rate ratio less than one indicates higher diagnostic confidence level, and rate ratio greater than one indicates lower diagnostic confidence level

^*^Intercept corresponds to mean rate

^‡^Global *p*-value

Confidence was lowest for breast diagnoses followed by renal diagnoses but the difference between the two is not significant (*p* = 0.380). The adjusted analysis gives the effect of a predictor after adjusting for other factors, thus as renal cases were all considered difficult to report, and difficult to report cases have low diagnostic confidence scores, when you adjust for difficulty, it is not surprising that renal was not the lowest confidence speciality. Confidence was highest for GI diagnoses.

Findings were similar when LM and DP data were analysed separately. The only noticeable difference was within the DP-only analysis, where pathologist’s overall reporting experience failed to reach significance (*p* = 0.083). Pathologist’s DP reporting experience was also found non-predictive of confidence (*p* = 0.78). This may be attributed to relatively few years of routine DP reporting experience by most pathologists but also to the lack of correlation between years of pathologists’ reporting experience and the ability to make a diagnosis of certain lesions using either diagnostic modality.

### Lowest confidence cases

There were 35 diagnoses where the pathologist had rated their diagnostic confidence as 1–3. This was split across 31 cases (in some instances, the pathologist gave a low score on both LM and DP). Of these 31, 14 were breast cases (2.3% of 608), 3 were GI (0.5% of 607), 6 were skin (1.0% of 609), and 8 were renal (4.0% of 200).

Only six (out of 16) pathologists contributed to the 35 low confidence diagnoses scores, despite the cases being split across all four specialities. It could be postulated that this is due to these pathologists having less experience; however, the results demonstrate a non-monotonic relationship between experience and confidence. Therefore, this relatively small group of pathologists contributing all the low confidence cases may be due to other factors, including individual variation in self-scoring.

#### Low confidence and case quality

Diagnostic confidence can be affected by the quality of glass slides or WSI. In these low confidence cases, several pathologists commented on the quality. For the LM low confidence cases, quality concerns included marginal biopsies, poor IHC, and a faded section. With DP cases, similar quality concerns were raised, but additional quality issues due to digitisation were also reported including scanned slides out of focus, poor quality or not high enough resolution. This suggests that overcoming these quality control issues, as could be done in practice, would increase reporting confidence.

#### Low confidence and case difficulty

The majority of the 31 low confidence cases (58.1%) were classified as difficult to report, in keeping with the notion that confidence falls in complex cases (Table 2).

**Table 2:** Details of the 31 low confidence cases. This lists all cases where a low confidence score was given (scores 1–3), along with the difficulty level, the confidence score by the same pathologist in the other diagnostic modality and the ground truth diagnosis (diagnostic confidence is given as 1–7 in which 1 is the lowest)

Specialty	Difficulty level	Ground truth diagnosis	Diagnostic confidence	
			LM	DP
Breast	Difficult	B3 without atypia B5a, DCIS low grade B5a, Encysted papillary carcinoma	**2** **2** **3**	7 4 6
		B5b, Invasive lobular carcinoma	**3**	6
		No metastatic malignancy. Lymphoid neoplasm requiring haematopathology review	7	**1**
	Moderate	B3 without atypia	**2**	7
	Routine	B1 normal A = B1, B = B2 B2	6 **1** 6	**2** **1** **1**
		B2	5	**3**
		B2 Sclerosing adenosis/sclerosed fibroadenoma	**1**	5
		B2 Fibroadenoma	**1**	7
		B5a	**3**	4
		B5b	**3**	4
GI	Moderate	pT4aN0R0	7	**3**
	Routine	Mild chronic gastritis, hyperplastic polyp	7	**3**
		Tubular adenoma with low-grade dysplasia	**1**	7
Skin	Difficult	Histiocytoma	**1**	**1**
		Erythema Multiforme likely to drug reaction	4	**1**
		Psoriasiform drug eruption	7	**3**
		Squamous cell carcinoma	**3**	7
		Spitz nevus	**3**	7
	Moderate	Lentigo maligna	**2**	**1**
Renal	Difficult	Focal segmental glomerulosclerosis/hypertensive glomerulosclerosis	4	**2**
		Thrombotic microangiography (TMA)	6	**3**
		No evidence of rejection, C4d negative	**3**	5
		IgA nephropathy	6	**1**
		Segmental necrotising glomerulonephritis—IgA out of focus on digital therefore unsuitable for diagnosis	7	**1**
		FSGS, all comments need EM to explore for LCPT (light chain proximal tubulopathy)	**2**	**3**
		IgA Nephropathy	5	**3**
		Lupus nephritis, class V	5	**1**

The GT diagnoses for these cases show some that are known to be difficult diagnostic areas within each speciality. For example, in the breast, there were rarer diagnoses including encysted papillary carcinoma and a lymphoid neoplasm, as well as B3 lesions which are a known area of diagnostic complexity. In the skin, there were also challenging areas including melanocytic lesions such as Spitz naevus and lentigo maligna and inflammatory skins such as erythema multiforme and a psoriasiform drug reaction. However, there were also some surprising diagnoses that are commonly reported. These include tubular adenoma with low-grade dysplasia, squamous cell carcinoma and fibroadenoma, for which no comments on poor quality were made. In several cases, pathologists commented that they would like to do further work before diagnosing such a case, including IHC and reviewing the case with colleagues. This is something that would be done in practice so likely to improve confidence beyond what was reported.

#### Low confidence and diagnostic discrepancy

In one-third (33.3%) of DP and half (52.9%) of LM low confidence diagnoses, there was a CID compared to GT, which is substantially higher than the overall values of 4.7% for LM and 4.8% for DP (as reported in Supplementary Table 1), indicating that there were several instances where pathologists were uncertain about their diagnosis, which subsequently corresponded to a diagnostic error. In these cases, the low confidence is most likely based on the awareness of making a difficult diagnostic judgement call.

### Diagnoses with high confidence but clinically important differences

There were a total of 514 diagnoses with a CID compared to the GT and a confidence score of 7. Although this is a small number of the total 16,187 diagnoses (3.2%), across all study diagnoses, there was a total of 765 CID diagnoses, highlighting that the majority of these clinically important incorrect diagnoses actually had a confidence score of 7.

Of these 514 cases, 251 (48.8%) were made on LM and 263 (51.2%) were on DP, with 174 occurring in the breast (3.5% of breast diagnoses), 250 GI (5.1%), 87 skin (1.8%) and 3 renal (0.2%). The types of CID errors between the diagnosis and GT were classified as above.

In some breast and skin cases, multiple errors were attributed to a single diagnosis, e.g. the diagnosis contained both a grading and IHC error, meaning there were 531 error types attributed to these 514 diagnoses. Table 3 shows the spread of different error types seen across each speciality, showing that for the breast, skin and GI, the main error type was diagnostic errors, which include tumour typing errors.

**Table 3:** Comparison of the different types of high confidence errors across specialities

Error type	Breast errors (*n* = 189) *N* (%)	GI errors (*n* = 250) *N* (%)	Skin errors (*n* = 89) *N* (%)	Renal errors (*n* = 3) *N* (%)
Diagnostic error—including differences in tumour subtype	121 (64.0)	214 (85.6)	48 (53.9)	1 (33.3)
Error in tumour grading	13 (6.9)	0 (0)	0 (0)	0 (0)
Error in tumour staging	24 (12.7)	10 (4.0)	11 (12.4)	0 (0)
Error in IHC interpretation	18 (9.5)	0 (0)	0 (0)	0 (0)
Erroneous errors	9 (4.8)	15 (6.0)	10 (11.2)	0 (0)
Threshold difference between pathologists	0 (0)	10 (4.0)	8 (9.0)	2 (66.7)
Error in margin measurement/excision status	4 (2.1)	1 (0.4)	12 (13.5)	0 (0)

#### Types of high confidence diagnostic errors by speciality

##### Breast diagnostic errors

Supplementary Table 2 shows a subcategorisation of the different types of breast errors. Excluding errors related to tumour subtyping (in which a malignant diagnosis was given but there were differences in the tumour type), the three most common types of breast diagnostic errors were B3 versus B2, (25.6%), B2 versus B3 (11.6%) and B1 versus B2 (9.9%). Another common source of errors was the presence or absence of atypia in B3 lesions.

Perhaps the most concerning error is a case where the GT diagnosis was B5b, but the pathologist diagnosis was B2. This happened in two instances (a single pathologist missed the same lesion on both LM and DP) which was missed lobular carcinoma.

An important error to note is the two instances where low-grade lymphoma was missed in a lymph node which instead was called normal. In practice, this case should have been seen by a lymphoma colleague or had a basic panel of IHC performed, but if the pathologist was highly confident in their diagnosis of a reactive lymph node, this may not have been instigated.

##### GI diagnostic errors

Missed high-grade dysplasia (the study pathologist reported low-grade dysplasia when the GT was high-grade dysplasia) was the most common error and accounted for 15.4% of the GI diagnostic errors (Supplementary Table 3). Overcalling dysplasia (where the GT was low-grade, but the pathologist reported high-grade) was also fairly common, accounting for 6.5% of GI diagnostic errors.

Another very common error was the differentiation between sessile serrated lesions (SSL) and hyperplastic polyps. In 14.0% of cases, the GT was SSL but the study pathologist called hyperplastic polyp, and in 6.1% of cases, the reverse was true (GT was hyperplastic, but study pathologist called SSL). This is also known to be an area of diagnostic complexity between pathologists.

Finally, a common diagnostic error was missing microorganisms. Missed microorganisms included Helicobacter pylori, Spirochetosis and Candida.

##### Skin diagnostic errors

The most common diagnostic discrepancies within skin pathology had to do with the subtyping of basal cell carcinomas (BCC) (Supplementary Table 4). This included both missing a high-risk subtype, e.g. infiltrative or overcalling a high-risk subtype, with this accounting for almost 40% of skin diagnostic errors, although this is likely due to the high frequency of BCC in the data. There were a few cases of benign versus malignant melanocytic lesions (Spitz naevus versus melanoma, benign naevus versus lentigo maligna for example) which is known to be a difficult area of skin pathology.

##### Renal diagnostic error

There was a single renal diagnostic error, with a GT diagnosis of no evidence of rejection, but a given diagnosis of borderline changes of T-cell-mediated rejection.

## Discussion

We have shown that, across 16 pathologists from six different NHS trusts and with varying levels of consultant experience, pathologists are generally highly confident in their diagnoses.

Our results demonstrate high confidence on both LM and DP, with overall diagnostic confidence only nominally higher for LM than DP. This is encouraging, particularly given the fact that most pathologists in the study had little or no experience of using DP in routine practice. It supports the wider introduction of DP into diagnostics, and we postulate that with time and further DP use, this small difference is likely to disappear.

In general, confidence was lower in cases where diagnostic discrepancies existed between the pathologist’s diagnosis and GT, suggesting pathologists are often aware of diagnostic difficulties in these error cases. It is reasonable to assume that in practice, this uncertainty would lead them to seek colleague review or further work to improve diagnostic accuracy. Confidence was also lower in cases where the pathologist changed their diagnosis between LM and DP, and so highlighting the issue of intra-pathologist variation. Unsurprisingly, the confidence level fell as case difficulty increased.

Interestingly, there was no correlation between the overall years reporting experience and confidence on LM or DP, with pathologists with mid-level experience seeming to be the least confident reporting overall. This does not match the suggestion in the literature that a lack of experience affects a pathologist’s ability to make a confident decision [2].

Analysis of the lowest confidence cases found that these were generally known difficult areas such as melanocytic lesions, inflammatory skins and rare breast lesions. However, surprisingly, low confidence was also seen, albeit rarely, in some common lesions such as tubular adenomas with low-grade dysplasia and squamous cell carcinoma in the skin, indicating that occasionally even commonly encountered lesions cause difficulties for the pathologist. Pathologists identified that quality issues both on LM and DP contributed to low confidence in selected cases and suggested that in practice they would seek further work, an indication that limitations imposed by the study environment did not fully reflect routine practice.

Analysis of the high confidence but incorrect cases found that these were predominantly diagnostic errors. Many of these errors were in areas with known diagnostic complexity such as B3 breast cancer screening lesions and large bowel SSLs, but we have shown that they can occur across the spectrum of conditions seen in each specialty. The rate of high confidence but incorrect diagnoses was lowest in renal (0.2% of renal diagnoses), potentially because renal is a known area where precise clinical correlation and further investigations (such as electron microscopy, which was not utilised in this study) are often essential to formulating a diagnosis.

It should come as no surprise that, in an interpretive discipline such as histopathology, opinions between pathologists will occasionally vary. In fact, although it may be easier to demonstrate and measure this difference in image-based diagnostic specialties, the same phenomenon will affect medicine more generally. Nevertheless, the detection and mitigation of such errors are difficult, and this remains an area where pathologists should consider what additional steps may need to be taken. The data presented here reinforces the importance of quality measures which aim to standardise objective opinion. Steps such as recognising diagnostic entities where a second opinion is needed or quality assurance schemes which assess the difference in defining tumour size, type, grade or levels of invasion are all important in reminding pathologists how their own opinion may differ from their peers.

## Conclusion

This large dataset provides a quantifiable understanding of pathologists’ confidence when reporting. Pathologists are slightly more confident on LM than DP, but the difference is small and likely reflects less familiarity with DP. The study shows lower diagnostic confidence is associated with difficult to report cases and cases with known inter- and intra-pathologist variation, but not with the level of pathologist experience. In many cases, low confidence can be addressed in practice through additional work and colleague review. High confident diagnoses that varied from the GT diagnosis were not insignificant and indicate that pathologists need to remain alert to diagnostic challenges and fully engage with professional development and quality assurance process where possible, in order to reduce as far as possible any negative impact on patient care.

## Supplementary Information

Below is the link to the electronic supplementary material.

Supplementary file1 (DOCX 21 KB)

Supplementary file2 (DOCX 22 KB)

Supplementary file3 (DOCX 24 KB)

Supplementary file4 (DOCX 22 KB)

## Data Availability

Data from this study is stored in the PathLAKE data lake and is available for further research. Applications for access to the data should be made to the PathLAKE Access Committee via the PathLAKE website.
