# Supplementary material for: What factors influence cellular pathologists’ confidence in case reporting?
Source: Virchows Arch. 2024 Aug 17;486(6):1165–73. doi: 10.1007/s00428-024-03899-1 (PMC12214028; doi:10.1007/s00428-024-03899-1)
Supplement: Supplementary file 1 — Supplementary file1 (DOCX 21 KB) [file 428_2024_3899_MOESM1_ESM.docx]

What factors influence cellular pathologists' confidence in case reporting? Virchows Archiv. H Evans et al. Corresponding author: [harriet.evans4@nhs.net](mailto:harriet.evans4@nhs.net)

***Supplementary table 1: Level of agreement between diagnoses***

| **Characteristic** | **Reports, n (%)** | |
| --- | --- | --- |
|  | **LM** | **DP** |
| Diagnosis same as GT?  Complete agreement  Clinically unimportant difference  Clinically important difference | 7210 (89.1)  507 (6.3)  377 (4.7) | 7173 (88.6)  532 (6.6)  388 (4.8) |
| LM and DP diagnoses same?  Complete agreement  Clinically unimportant difference  Clinically important difference | 7352 (90.8)  416 (5.1)  326 (4.0) | 7353 (90.9)  416 (5.1)  324 (4.0) |

*DP- Digital pathology, GT- Ground truth, LM- light microscopy*
