# Supplementary material for: What factors influence cellular pathologists’ confidence in case reporting?
Source: Virchows Arch. 2024 Aug 17;486(6):1165–73. doi: 10.1007/s00428-024-03899-1 (PMC12214028; doi:10.1007/s00428-024-03899-1)
Supplement: Supplementary file 2 — Supplementary file2 (DOCX 22 KB) [file 428_2024_3899_MOESM2_ESM.docx]

What factors influence cellular pathologists' confidence in case reporting? Virchows Archiv. H Evans et al. Corresponding author: [harriet.evans4@nhs.net](mailto:harriet.evans4@nhs.net)

***Supplementary table 2: Breast high confidence diagnostic errors***

| **Type of diagnostic error** (GT vs study pathologist’s diagnosis) | **Number of occurrences (% of total breast diagnostic errors)** | **Comments** |
| --- | --- | --- |
| B1 vs B2 | 12 (9.9) |  |
| B2 vs B1 | 7 (5.8) |  |
| B2 vs B3  *B2 vs B3 without atypia*  *B2 vs B3 with atypia* | 14 (11.6)  3  11 |  |
| B2 vs B4 | 1 (0.8) |  |
| B3 vs B2  *B3 without atypia vs B2*  *B3 with atypia vs B2* | 31 (25.6)  22  9 |  |
| B3 vs B4 | 4 (3.3) |  |
| B3 vs B5a | 1 (0.8) |  |
| B3/B4 vsB5b | 2 (1.7) |  |
| B3/B4 vs B5c | 1 (0.8) |  |
| B3 without atypia vs B3 with atypia | 7 (5.8) |  |
| B3 with atypia vs B3 without atypia | 8 (6.6) |  |
| B5a vs B3 | 2 (1.7) |  |
| B5a vs B4 | 1 (0.8) |  |
| B5b vs B2 | 2 (1.7) |  |
| Malignant vs benign | 2 (1.7) | Case of low-grade lymphoma vs reactive lymph node |
| Melanoma vs carcinoma | 1 (0.8) | Case of lymph node metastasis |
| Absence vs presence of residual DCIS | 1 (0.8) |  |
| Tumour subtyping error | 24 (19.8) | Range of misclassifications (mixed vs pure tumours, lobular vs NST) |

*DCIS: Ductal carcinoma in situ, NST: No special type.*

*In the table the GT diagnosis is given first, followed by the given diagnosis by the study pathologist, for example B1 vs B2 means the GT diagnosis was B1, but the study pathologist called the case B2.*
