# Supplementary material for: What factors influence cellular pathologists’ confidence in case reporting?
Source: Virchows Arch. 2024 Aug 17;486(6):1165–73. doi: 10.1007/s00428-024-03899-1 (PMC12214028; doi:10.1007/s00428-024-03899-1)
Supplement: Supplementary file 3 — Supplementary file3 (DOCX 24 KB) [file 428_2024_3899_MOESM3_ESM.docx]

What factors influence cellular pathologists' confidence in case reporting? Virchows Archiv. H Evans et al. Corresponding author: [harriet.evans4@nhs.net](mailto:harriet.evans4@nhs.net)

***Supplementary table 3: GI high confidence diagnostic errors***

| **Types of diagnostic error**  (where applicable, GT vs study pathologist’s diagnosis) | **Number of occurrences**  **(% of total GI diagnostic errors)** |
| --- | --- |
| **Incorrect grading of dysplasia** | **67 (31.3)** |
| Missed low grade dysplasia | 8 (3.7) |
| Missed high grade dysplasia (HGD called LGD) | 33 (15.4) |
| Low grade dysplasia overcalled high grade dysplasia | 14 (6.5) |
| Indefinite for dysplasia vs no dysplasia | 4 (1.9) |
| No grade of dysplasia given for polyp | 4 (1.9) |
| Adenocarcinoma vs low grade TA | 4 (1.9) |
| **Missed metaplasia** | **8 (3.7)** |
| Missed intestinal metaplasia | 6 (2.8) |
| Overcalled intestinal metaplasia (when not present) | 1 (0.5) |
| Missed gastric metaplasia | 1 (0.5) |
| **Difference in types of polyp** | **60 (28.0)** |
| SSL vs hyperplastic polyp | 30 (14.0) |
| Serrated polyp with dysplasia vs hyperplastic polyp | 3 (1.4) |
| Hyperplastic polyp vs SSL | 13 (6.1) |
| TA/TVA vs Hyperplastic polyp | 1 (0.5) |
| TA/TVA vs SSL | 1 (0.5) |
| SSL vs TA/TVA | 8 (3.7) |
| Serrated polyp NOS vs TA/TVA | 1 (0.5) |
| Fundic gland polyp vs hyperplastic polyp | 3 (1.4) |
| **Polyp vs no polyp** | **19 (8.9)** |
| Normal vs hyperplastic polyp | 1 (0.5) |
| Hyperplastic polyp vs normal | 13 (6.1) |
| Hyperplastic polyp vs mild chronic gastritis | 2 (0.9) |
| TA/TVA vs active colitis | 1 (0.5) |
| TA/TVA vs focal atypia no polyp | 1 (0.5) |
| TA/TVA vs normal | 1 (0.5) |
| **Disease vs non disease** | **17 (7.9)** |
| Active inflammation vs normal | 1 (0.5) |
| No inflammation vs active inflammation | 1 (0.5) |
| IBD vs normal | 1 (0.5) |
| Missed amyloid | 4 (1.9) |
| Missed coeliac disease | 6 (2.8) |
| Missed neuroendocrine tumour | 2 (0.9) |
| No GIST vs suspicious for GIST | 2 (0.9) |
| **Barrett's** | **15 (7.0)** |
| Barrett's vs No Barretts | 13 (6.1) |
| No Barretts vs Barrett's | 2 (0.9) |
| **Other** | **28 (13.1)** |
| Missed small objects (microorganisms) | 19 (8.9) |
| IBD vs features not sufficient for IBD | 3 (1.4) |
| Error in tumour typing | 3 (1.4) |
| Presence vs absence of intra-mural venous invasion | 3 (1.4) |

*GIST: Gastrointestinal stromal tumour, HGD: high grade dysplasia, IBD: Inflammatory bowel disease, LGD: low grade dysplasia, NOS: not otherwise specified, SSL: sessile serrated lesion, TA: tubular adenoma, TVA: Tubulovillous adenoma.*

*In the table the GT diagnosis is given first, followed by the given diagnosis by the study pathologist.*
