# Supplementary material for: What factors influence cellular pathologists’ confidence in case reporting?
Source: Virchows Arch. 2024 Aug 17;486(6):1165–73. doi: 10.1007/s00428-024-03899-1 (PMC12214028; doi:10.1007/s00428-024-03899-1)
Supplement: Supplementary file 4 — Supplementary file4 (DOCX 22 KB) [file 428_2024_3899_MOESM4_ESM.docx]

What factors influence cellular pathologists' confidence in case reporting? Virchows Archiv. H Evans et al. Corresponding author: [harriet.evans4@nhs.net](mailto:harriet.evans4@nhs.net)

***Supplementary table 4: Skin high confidence diagnostic errors***

| **Type of diagnostic error** (GT vs study pathologist’s diagnosis) | **Number of occurrences**  **(% of total skin diagnostic errors)** | **Type** |
| --- | --- | --- |
| Low risk vs high-risk BCC | 12 (25.0) | Upgraded BCC subtype |
| High risk vs low-risk BCC | 7 (14.6) | Downgraded BCC subtype |
| AK vs SCC | 3 (6.3) | In situ vs invasive malignancy |
| SCC vs AK | 2 (4.2) | Invasive vs in situ malignancy |
| SCC vs Keratoacanthoma | 1 (2.1) | Incorrect type of malignant tumour |
| SCC vs BCC | 6 (12.5) | Incorrect type of malignant tumour |
| SCC vs melanoma | 1 (2.1) | Incorrect type of malignant tumour |
| BCC vs SCC | 1 (2.1) | Incorrect type of malignant tumour |
| Bowen's disease vs AK and BCC | 2 (4.2) | In situ vs invasive malignancy |
| Bowen's disease vs statis dermatitis | 1 (2.1) |  |
| Benign Intradermal/Compound Naevus vs lichenoid keratosis/inflammatory reaction and lentigo maligna | 3 (6.3) | Upgraded benign naevus to in-situ melanocytic lesion |
| Spitz naevus / Reed naevus vs melanoma | 3 (6.3) | Upgraded benign naevus to melanoma |
| Melanoma vs blue naevus | 1 (2.1) | Malignant vs benign melanocytic lesion |
| Metastatic melanoma vs benign lymph node | 3 (6.3) | Missed metastatic disease |
| Dermatofibroma vs DFSP | 2 (4.2) |  |

*AK: actinic keratosis, BCC: basal cell carcinoma, DFSP: Dermatofibrosarcoma protuberans, SCC: squamous cell carcinoma.*

*In the table the GT diagnosis is given first, followed by the given diagnosis by the study pathologist.*
